# Integration of Nanobots Into Neural Circuits As a Future Therapy for Treating Neurodegenerative Disorders

**DOI:** 10.3389/fnins.2018.00153

**Published:** 2018-03-21

**Authors:** Arthur Saniotis, Maciej Henneberg, Abdul-Rahman Sawalma

**Affiliations:** ^1^Biological Anthropology and Comparative Anatomy Unit, School of Medicine, University of Adelaide, Adelaide, SA, Australia; ^2^Institute of Evolutionary Medicine, University of Zürich, Zurich, Switzerland; ^3^Palestinian Neuroscience Initiative, Al-Quds University, Beit Hanina, Palestine

**Keywords:** nano-neural symbiosis, endomyccorhiza, communicative coupling, post-surgical problems, cerebrospinal fluid

## Abstract

Recent neuroscientific research demonstrates that the human brain is becoming altered by technological devices. Improvements in biotechnologies and computer based technologies are now increasing the likelihood for the development of brain augmentation devices in the next 20 years. We have developed the idea of an “Endomyccorhizae like interface” (ELI) nanocognitive device as a new kind of future neuroprosthetic which aims to facilitate neuronal network properties in individuals with neurodegenerative disorders. The design of our ELI may overcome the problems of invasive neuroprosthetics, post-operative inflammation, and infection and neuroprosthetic degradation. The method in which our ELI is connected and integrated to neuronal networks is based on a mechanism similar to endomyccorhizae which is the oldest and most widespread form of plant symbiosis. We propose that the principle of Endomyccorhizae could be relevant for developing a crossing point between the ELI and neuronal networks. Similar to endomyccorhizae the ELI will be designed to form webs, each of which connects multiple neurons together. The ELI will function to sense action potentials and deliver it to the neurons it connects to. This is expected to compensate for neuronal loss in some neurodegenerative disorders, such as Alzheimer's disease and Parkinson's disease.

## Introduction

Recent neuroscientific research indicates that the human brain is becoming altered by technological devices. Cognitive enhancement therapies are now underway for remediation of neurocognitive and neurodegenerative deficits in individuals with schizophrenia and paraplegia/tetraplegia (Hogarty and Greenwald, 2006; Aflalo et al., 2015; Wojtalik et al., 2016). Brain-machine interfaces (BCI) have been successful in generating physical movement from brain signals which with training enables navigating a computerized wheelchair (Bouton et al., 2016; Rajangam et al., 2016; Sharma et al., 2016). This may path the way for future therapeutic BCIs enabling individuals to use their limbs and produce speech (Shih et al., 2012). One of the latest neuroprosthetic (Ipsihand) allows a paralyzed hand to move. The device detects electrical brain signals of the wearer's intention causing subsequent movement of the hand (Bundy et al., 2017). Recently, a 12-month BMI gait neurorehabilitation program was used on 8 spinal cord paraplegic patients with the aim of restoring movement. The program utilized ongoing visual-tactile feedback and virtual reality in conjunction with a tactile exoskeleton and EEG-controlled robotic actuators during walking (Donati et al., 2016). After the completion of the program all 8 patients reported somatic sensory improvements in several dermatomes, and regaining of voluntary motor control in key muscles below the level of the spinal cord lesion (Donati et al., 2016). The results indicated the possibility of neurological recovery due to long term BMI usage which fosters neuroplasticity (Donati et al., 2016). Furthermore, genome editing shows promise in remedying genetic diseases and neurological disorders. Recently, genome editing protein (-27)GFP-Cre was delivered *in vivo* into a mouse brain via bioreducible lipid nanoparticles for DNA recombination (Wang et al., 2016).

Using the Nanotechnology for helping patients with neurodegenerative disorders is a natural step forward in the world of nanotechnology. Neurodegenerative disorders, such as Parkinson's disease or Alzheimer's disease, cause significant burden on the health care system, and affects the quality of life significantly (Schrag et al., 2000; Hebert et al., 2003). This type of disorders is associated with neural degeneration, causing significant and pathological alterations of nerve conduction that manifest as a neurological disorder. For example, In Parkinson's disease there is a degeneration of the medium spiny neurons in the substantia nigra, which reduces stimulation of dopamine neurons in the striatum which causes an imbalance between the direct and indirect pathways that are connected to the motor activity of the person (Calabresi et al., 2014).

From a neurosurgical viewpoint previous and current neuroprosthetic research has essentially followed a mechanistic approach to cortical function which is suitable for understanding sensory and motor processes. However, such an approach is not feasible for comprehending associative cortical functions dealing with higher order cognition. This is because higher order cognitive functions work on multiple intersecting and widely dispersed neural networks. Our understanding of how these networks function and co-operate in cortical and sub-cortical areas is still insufficient, as are their evolutionary substrates. Another issue confronting neurosurgeons has been the surgical implantation of a neuroprosthetic device which is fraught with various medical challenges. These have included the precise surgical placement of a neuroprosthetic device in order to optimize its functional capabilities. Previous studies indicate that the type of neurosurgical implantation approach can influence neurobiological performance (Nicolelis et al., 2003; Liu et al., 2015). It has also been noted that speed of insertion of a neuroprosthetic device may affect neurobiological response (Biran et al., 2007). Furthermore, chronic electrode implants undergo degradation. This degradation considerably reduces recording activity in non-human primate models (Schwartz, 2004). Signal attenuation of chronic implanted electrodes is primarily caused by host brain tissue response to a foreign object which can lead to microglial activated inflammation and glial scarring (Nicolelis et al., 2003; Polikov et al., 2005), resulting in displacement of proximal neurons from the implanted electrode's surface. Another reported post-neurosurgical response to prosthetic devices has been rupture of the blood brain barrier resulting in infiltration of cytokines, platelets and leucocytes (Polikov et al., 2005) into the CNS, increasing inflammatory response and subsequent edema (Schmidt et al., 1993). We have developed the idea of a “*endomyccorhizae*-like interface” (ELI) nanocognitive device as a new kind of future neuroprosthetic which aims to facilitate neuronal network properties in individuals with neurodegenerative disorders. The design of our ELI may overcome the problems of invasive neuroprosthetics, post-operative inflammation and infection and neuroprosthetic degradation.

**Figure 1:**
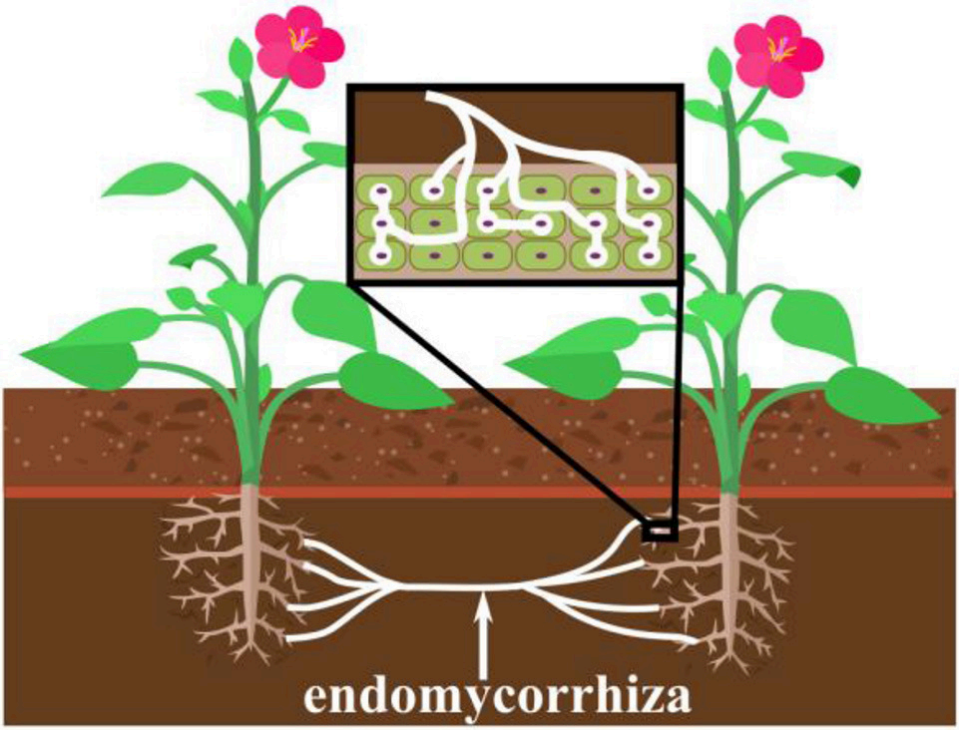
The concept of the *endomycorrhiza* and its mechanism for penetration into plant root cells of the plants' roots, and how it can simultaneously connect two different plants. This action is believed to transfer biological properties between the plants, such as the immune response of one plant to the other.

The method in which our ELI is connected and integrated to neuronal networks is based on a mechanism similar to *endomyccorhizae* which is the oldest and most widespread form of plant symbiosis (Brundrett, 2002). For over 400 million years *endomyccorhizae* has formed ~80 of terrestrial plant/fungi interactions (Wang and Qiu, 2006). During *endomyccorhizae*, bundles of finger like fungal extensions called mycelium (*hyphae*), also known as *shiro*, penetrate the plant root where they form arbuscules (branching structures) spores and vesicles within the root cell structure (Figure 1). Due to its integrative method mycelium facilitates mineral uptake of potassium, nitrogen, zinc and copper to the plant root, protecting against infection and conferring plant fitness (Sikes et al., 2010; Tahat et al., 2010). Another significant feature of *endomyccorhizae* is how mycelium interacts with other soil organisms in a beneficial manner (Tahat et al., 2010). *Endomyccorhizae* may also lead to anatomical changes in plant root morphology via mycelium colonization (Tahat et al., 2010). Mycelium colonization can increase plant growth and reduce pathogen invasion (Tahat et al., 2010).

We propose that the principle of *endomyccorhizae* could be relevant for developing a crossing point between the ELI and neuronal networks. Similar to *endomyccorhizae* the ELI will be designed to form webs, each of which connects multiple neurons together. This is necessary because neurons are connected with many other neurons so that a single neuron can be involved in several different neuronal circuits. The ELI will function to sense action potentials and deliver it to the neurons it connects to. This is expected to compensate for neuronal loss in some neurodegenerative disorders, such as Alzheimer's disease and Parkinson's disease.

## How the ELI would work

**Figure 2:**
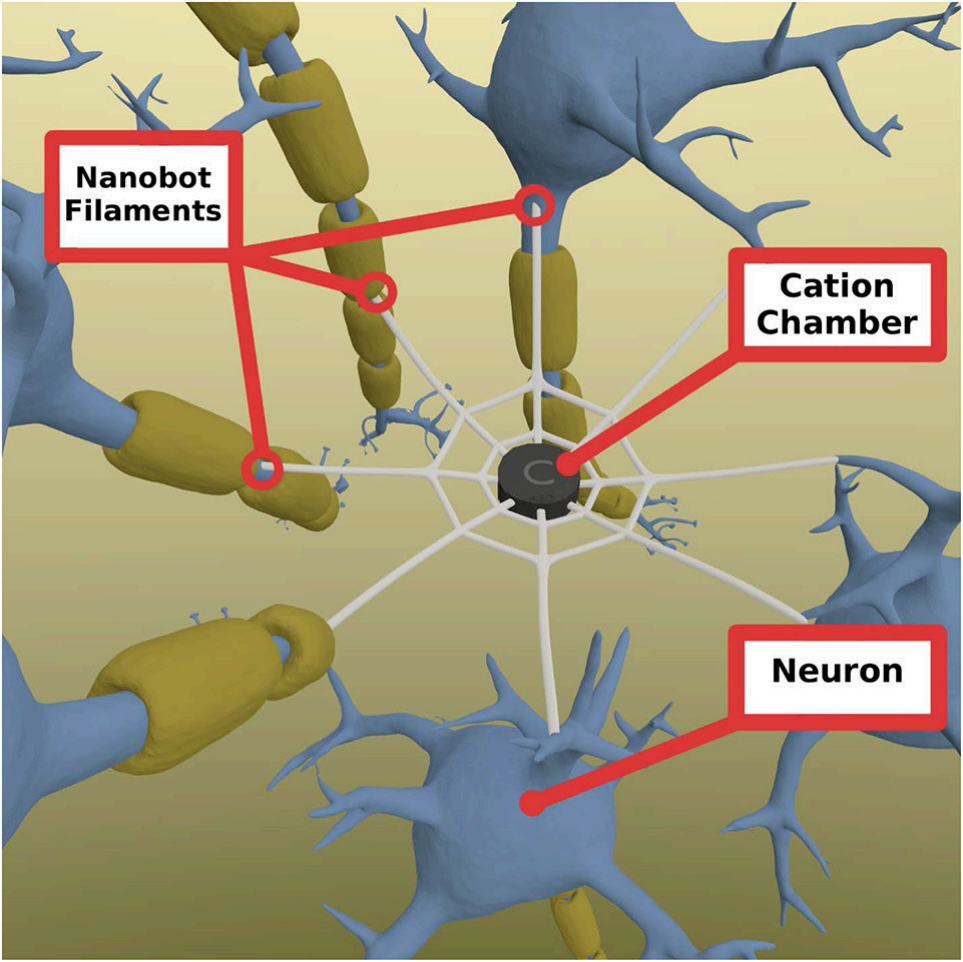
Nanobot mesh consisting of a central chamber of cations that allow cation to enter selectively and is able to store them for a short period of time if needed. These cations can be released at when the signal comes from any of the connected neurons through the fibers. The fibers are radial extensions from the mesh that connect to other neurons and penetrates them to open inside the cell. They are able to sense the actiton potential and send a signal back to the champer to open the chamber gates to allow cations to travel to each of the connected neurons. Chamber gates (not shown) are gates connecting the chamber and the fibers that open when a signal is received. Note: the diameter of the fibers is greatly enhanced for visual presentation purposes.

The mechanism of the ELI is that it would seek the perikarya of neurons which show increased electric activity to attach to them, or to enter them. The ELI will consist of cation chamber containing cations extracted from the surrounding environment. The outbranching mesh fibers have a special type of tip that is able to penetrate the cell membrane of a neuron (Figure 2).

When the action potential reaches the tip of the device that is inserted into the neuronal membrane, the influx of cations can affect the charge of the inside of the fiber, making it more positive. This change in charge can be transmitted to the chamber, activating it to release cations to all other connected neurons. As it is important to be optimal in the use of the stored cations, the flow of cations should be timely controlled in a way that mimics normal action potential timing, while it should be strong enough to pass the threshold of the neuronal membrane for it to trigger an action potential and propagate it to the end of the axon. In order to allow for the control of the rate of cation release, the chamber gates should be designed so as to mimic the natural slow-closing sodium channels. The penetrability of the ELI should be very specific to the neuronal axons, somas and/or dendrites. This may be feasible by exploiting the biochemical properties of neuronal membranes. Therefore, in theory, we may be able to target specific neurons in specific brain regions. The ELI could be engineered to have ligand-like properties that attach to specific receptors on the axonal membranes, using them to penetrate the membrane, thereby achieving connectivity. Consequently, the ELI will have the potential to sense neurotransmitter potentials in cell membranes. In that sense ELIs are functionally dependent on the action potential passing through the axon of the cell. ELIs functions only to propagate the action potential and send it to the neurons it connects to, or it enhances the rate of the action potential they receive. Thus, no modification of the normal functions of the neuron, it is rather a method of regaining the function of the disrupted neural pathway (e.g., in individuals with neurodegenerative disorders).

The device should be made specific to the cells it targets. This can be done in two levels. The first level includes engineering the device's tips so that they have binding specificity, like ligands, to specific membrane proteins that are different according to cell type. This way the device will prefer binding to this required type. The other level is electrophysiological: The device should be engineered so that it sends the send action potentials at a speed that is just slower than the normal range of the speed of the targeted tissue. This way, if the neurons are healthy, the device sends the signals from the pre-device neurons to the post-device neurons in the refractory period of the post-device neurons, which will not induce any unwanted extra activation of the post-device neuron. If, however, the pre-synaptic neurons are degenerated, the action potential induced by the device will reach the next neurons in a resting state.

Permanent presence of ELIs would require their energy supply to derive from biological processes of surrounding tissues such like thermal energy or fluid flow, electrolytes in neurons or glial cells (Senthilnathan et al., 2016), or even ATP (Yadav et al., 2015).

## Delivery of ELI into brain tissue

Currently, most nanotechnological devices are delivered to various cells in the body via bloodstream (Felfoul et al., 2016). Since the existence of blood-brain barrier may make bloodstream delivery of nanoparticles to the brain impractical, an ELI can be introduced directly into the cerebrospinal fluid. This would be accomplished via injecting the ELI through the roof of the orbit with a fine needle. Since the meningeal structures line the endocranial side of the roof of the orbit, this is an accessible route to penetrate into the subarachnoid space with minimal damage to anatomical structures. Upon piercing the roof of the orbit the needle would be inserted ~1 mm into the subarachnoid space, thus entering the cerebrospinal fluid. The ELI should not float or sink in the arachnoid space. Therefore, the specific gravity of the ELI should be ~1.036 grams per milliliter. We envisage that a ELI would move in the CSF, possibly with the use of a propeller. Alternately, biotechnologists could make ELI to travel within the entire CNS in order to attach to its destined neuronal site(s). Additionally, an outside probe could be used in order to shut down any ELI that is not in a required area.

## Immune response

The ELI will be foreign to the body, and the immune system is built to attack foreign agents like this. It is possible that the immune system may form a capsule that might disrupt the function of the ELI (Zhang et al., 2013). Although we do not fully understand the mechanisms of immune system and how to selectively inhibit it, researchers have used multiple methods to “trick” the immune system into not attacking foreign bodies. These methods have been suggested for medical devices of various sizes. Some used cationic coating to suppress immune response (Ma et al., 2011). They provided evidence that poly (β-amino alcohols) have the ability to weaken the response to foreign bodies. Other researchers suggest that zwitterionic hydrogels, which are hydrogels whose molecules contain two functional groups with opposite charges, can inhibit encapsulation and immune response to the foreign body for at least 3 months (Zhang et al., 2013). On the other hand, Veiseh et al. (2015) showed that smaller-sized devices decrease immune response compared to larger ones. These methods, and others, should be further tested in the ELI specifically to determine which to use and how effective it is prior to using the ELI in humans.

## Feasibility of ELI

More understanding of the processes underlying multiple intersecting and widely dispersed neural networks will be vital for engineering a ELI in order to modify neuronal network properties in patients. Whether such a ELI is feasible is probably best answered by the current research on hippocampal prosthetics for memory replacement and recovery in subjects with Alzheimer's disease (AD) and cerebral trauma. So far hippocampal prosthetics have been used on rodents. In one animal study (rodents) the results indicated that a multi input multi output (MIMO) model when applied to CA3 and CA1 hippocampal areas assisted in memory functioning (Berger et al., 2012).

## Conclusion

We propose that the ELI is a novel neuroprosthetic design whose method of connectivity and integration with neuronal networks is informed by *endomyccorhizae*. Interfacing to the nervous system has been a considerable problem because of inflammation and subsequent infection, degradation of the neuroprosthetic and unwanted movement of the neuroprosthetic. Furthermore, recent innovations in medical neurology, genetic editing, and nano-neuroscience are making the construction of such an ELI feasible to treat neurodegenerative conditions.

## Author contributions

AS: Initiated topic and investigation into NCE. Organized theoretical content, diagrams, and edited article with other authors. MH: Co-developed idea of NCE. Contributed to theoretical analysis of article. Assisted in proofreading of article. A-RS: Contributed to theoretical analysis of article and created Figure 1.

### Conflict of interest statement

The authors declare that the research was conducted in the absence of any commercial or financial relationships that could be construed as a potential conflict of interest.
